# Systematic Literature Review and Meta-Analysis of Anti-Psychotic Use in Parkinson's Disease Psychosis

**DOI:** 10.1192/bjo.2024.701

**Published:** 2024-08-01

**Authors:** Christopher McKeown, Alberto Salmoiraghi

**Affiliations:** Betsi Cadwaladr University Health Board, Wrexham, United Kingdom

## Abstract

**Aims:**

Psychosis is a common neuropsychiatric symptom associated with Parkinson's disease (PD), with prevalence rates of up to 75%. Parkinson's disease psychosis (PDP) is associated with increased morbidity, caregiver burden, depression, poorer quality of life and progression of dementia. It has also been shown to be a strong predictive factor for long term care placement, and results in up to 71% increase in risk of mortality compared with PD patients free from psychotic symptoms. Use of antipsychotics for PDP is common, with up to 35% of PD patients prescribed at least one antipsychotic within 7 years of PD diagnosis. This systematic literature review aims to search, appraise and synthesise the best available and most up-to-date evidence for the use of antipsychotics in the treatment of PDP, and their effects on PD motor symptoms.

**Methods:**

We carried out a comprehensive literature review and meta-analysis following the PRISMA statement for systematic reviews.

**Results:**

Four studies investigated quetiapine, three investigated olanzapine, two investigated clozapine and a further two investigated pimavanserin. Both quetiapine and olanzapine showed no significant improvement for PDP over placebo, however meta-analysis of olanzapine groups showed significant motor worsening, UPDRS +2.89 (95% CI 1.22 to 4.56) compared with placebo. Clozapine showed a significant improvement in psychosis vs placebo in both studies, with a large effect size in their primary outcome measure; -0.82 (95% CI -1.37 to -0.26), -0.89 (95% CI -1.42 to - 0.36). Pimavanserin showed significant improvement in psychosis vs placebo -0.48 (95% CI -0.77 to -0.18). Quetiapine, clozapine and pimavanserin showed no significant worsening in motor scores vs placebo group.

**Conclusion:**

Although Olanzapine and Quetiapine are commonly used to treat psychotic symptoms in Parkinson's Disease, the only medication with robust evidence is Clozapine. This finding may have implications for service delivery.

